# Definite Treatment Delay With Neoadjuvant Chemotherapy and Longitudinal Monitoring by Circulating Tumor DNA for Advanced Cervical Cancer During Pregnancy: A Case Series and Literature Review

**DOI:** 10.1002/cnr2.70021

**Published:** 2024-11-07

**Authors:** Xiaoyu Zhang, Hong Yu, Kai Chen, Bo Ding, Yang Shen

**Affiliations:** ^1^ Department of Obstetrics and Gynecology, Zhongda Hospital Southeast University Nanjing China; ^2^ Novant Health Maternal Fetal Medicine Winston‐Salem North Carolina USA

**Keywords:** advanced cervical cancer, chemoradiotherapy, circulating tumor DNA, neoadjuvant chemotherapy, pregnancy

## Abstract

**Background:**

Previous studies mainly concentrate on neoadjuvant chemotherapy (NACT) for delivery delay in FIGO Stage IB1–IIIB cervical cancer during pregnancy to prevent early preterm delivery while not affecting maternal outcome.

**Case:**

Here, we described two pregnant patients with FIGO Stage IIIC cervical cancer about their diagnosis, treatment, and outcome. Both patients underwent cesarean delivery, left enlarged lymph node dissection, and longitudinal monitoring by circulating tumor DNA. Our study suggested that pregnant patient was completely response to NACT, which was confirmed by ctDNA monitoring, followed by left pelvic enlarged lymph node dissection during cesarean section and adjuvant chemoradiotherapy postpartum. The infant grew normally, without any evidence of chemotherapy‐related side effects after delivery.

**Conclusion:**

In pregnant women with advanced cervical cancer, longitudinal ctDNA monitoring might be able to evaluate maternal response to NACT and confirm if delivery delay to optimize fetal outcome would compacting the maternal outcomes or not. Cervical cancer may not transmit across the placental barrier and so it is safe for delayed delivery until fetal maturity in utero during pregnancy.

## 1 Introduction

Cervical cancer is one of the most frequent cancers during pregnancy, with an incidence ranging from 1.4 to 4.6 per 100 000 pregnancies [1, 2]. The standard therapy for nonpregnant women with locally invasive cervical cancer (FIGO Stage IB3–IVA) include concurrent chemoradiotherapy or neoadjuvant chemotherapy (NACT) followed by surgery [3]. Currently, the management of cervical cancer in pregnancy is still a challenge and lack of study. It mainly depends on multiple factors, such as the International Federation of Gynecology and Obstetrics (FIGO) stage, histologic features, and gestational age at the time of diagnosis. The optimal therapy for pregnant women with cervical cancer remains a challenge mainly due to the lack of methods to monitor and ensure clinicians and patients that delaying delivery for fetal maturity without affecting maternal outcomes. Previous study suggested that expectant management for early‐stage cervical cancer (FIGO Stage < IB1) diagnosed during pregnancy has been depicted since fetal lung maturity seemingly does not worsen the maternal outcome [1]. For pregnant women with FIGO Stage ≥ IB1, the best or standard management is challenging and still unclear. For pregnant women with FIGO Stage IB1–IIIB cervical cancer, previous studies suggested that NACT contributes to controlling the disease and deferring delivery until fetal viability [4, 5]. To date, as we know of, there is a lack of reported studies specifically detailing cases of pregnant women with FIGO Stage IIIC or higher cervical cancer who have successfully achieved the delay of delivery for fetal maturation through NACT without adversely affecting maternal outcomes.

Circulating tumor DNA (ctDNA) is a valuable component of circulating cell‐free DNA that carries genetic information from tumors. Analysis of ctDNA can provide information that is in accordance with that accessed from tumor tissue biopsies to some extent, enabling the identification of somatic mutations, polymorphic sequence variants, or gene fusions that are potentially helpful for diagnosis, prognosis, or monitoring the disease [6, 7]. Compared with tissue biopsy, ctDNA is less invasive and can offer a more comprehensive landscape of tumor heterogeneity [8]. In the era of precision oncology, next‐generation sequencing (NGS) based assays using liquid biopsy in ctDNA detection has been increasingly applied as a complimentary tool in clinical practice [9], but its application for pregnant patient is unknown [10].

Here, we present a case series of two pregnant women with FIGO Stage IIIC cervical cancer, highlighting the successful delay of definite treatment using NACT. This delay was achieved without compromising maternal outcomes, thanks to the monitoring of maternal ctDNA. In the first case, treatment was deferred until fetal maturity, while in the second case, it was postponed until scar healing, which was the first as we know of using in pregnant woman. The outcome is reported in comparison to other conventional monitoring tools.

## 2 Case Presentation

### 2.1 Patient 1

A 28‐year‐old gravida‐2 para‐0 female with a desired singleton pregnancy. She presented to The First People's Hospital of Lianyungang at 20 weeks and 4 days gestation (dated by her sure last menstrual period and confirmed by first trimester ultrasound) because of intermittent vaginal bleeding for 7 days on December 25, 2021. She denied previous history of chronic diseases or gynecological tumors. Cervical Thinprep cytologic test (TCT) was repeated at 21 weeks of gestation and showed suspicious squamous cell carcinoma. Transvaginal ultrasound showed a hypoechoic mass measuring 64 × 54 mm around the left iliac vessels and a cervical mass measuring 21 × 13 mm. The patient had normal cervical pap smear 2 months before pregnancy in local hospital where she had her prenatal care. The patient was transferred to Zhongda Hospital, a regional referring medical center, for further evaluation and management at 24 weeks of gestation on January 18, 2022. Magnetic resonance imaging (MRI) of the abdomen and pelvis revealed a viable intrauterine single pregnancy, cervical mass along with left parametrial involvement and multiple enlarged lymph node around the left iliac vessels. Cervical biopsy showed cervical invasive squamous cell carcinoma, moderately differentiated and the staging was IIIC based on the 2018 FIGO Staging System. The serum squamous cell carcinoma antigen (SCCA) value was 13.1 ng/mL (normal range 0–2.7 ng/mL), and human papillomavirus (HPV) test was positive for Type 16. ctDNA mean allele frequency (AF) by targeted NGS of 202 cancer‐related genes was 0.6%.

A multidisciplinary meeting was held composed of gynecologic oncologists, obstetricians, pediatricians, urologists, interventional radiologists, radiologists, radiation oncologists, pathologists, and ethical advisors. Options, risks, and benefits of severe preterm delivery with maternal therapy immediately versus delay delivery for fetal maturity with NACT during pregnancy followed by standard cervical cancer treatment postpartum monitored by ctDNA, SCCA detection, and MRI with possible risks of compromising maternal outcomes were reviewed with the patient and her husband. The patient and her husband declined severe preterm delivery but desired delay delivery.

**FIGURE 1:**
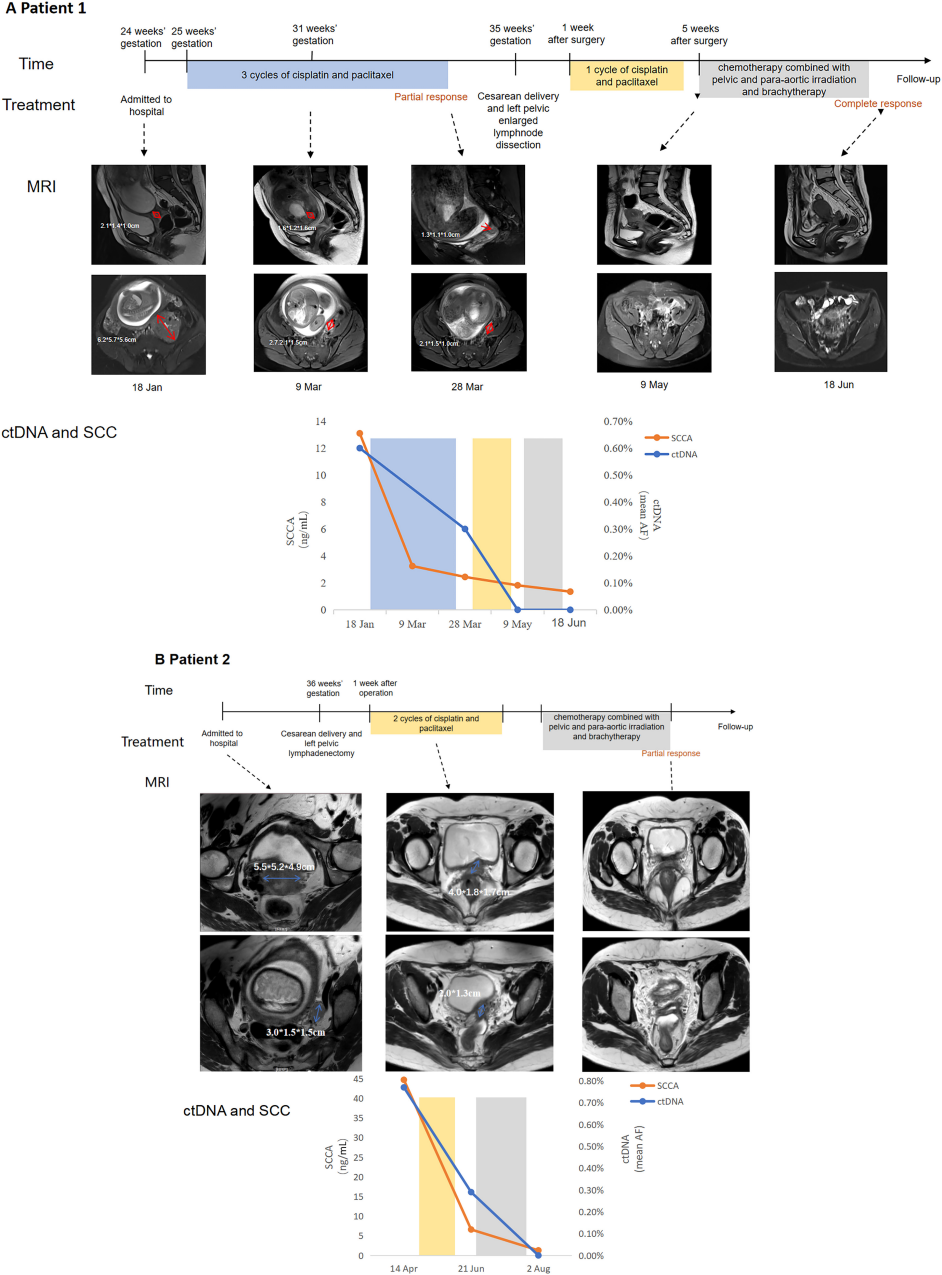
Clinical managements of two patients. Panel A and Panel B show the clinical courses of Patient 1 and Patient 2, respectively, timelines for treatments, detailed radiology results, ctDNA and SCC changes. MRI: magnetic resonance imaging; SCC: squamous cell carcinoma antigen; ctDNA: circulating tumor DNA.

At 25 weeks of gestation, NACT with three‐weekly cisplatin (70 mg/m^2^) and paclitaxel (175 mg/m^2^) was started as per protocol. After two cycles of NACT, due to lack of information from previous study, patient response to NACT was evaluated at 31 weeks of gestation by both MRI, which showed significant shrinkage of the tumor, and by SCCA, which showed markedly decreased to 3.25 ng/mL from initial level at 13.1 ng/mL. Given that the tumor appeared well response to NACT and the fetus already 31 weeks of gestation, the patient and her husband desired the third cycle of NACT and hoped to delay ctDNA detection until cesarean delivery for economic reasons. The third cycle of NACT was used at 33 weeks of gestation. At 35 weeks of gestation, 2 weeks after the last predelivery cycle of chemotherapy, the patient was delivered via cesarean delivery 2 days after antenatal corticosteroid. Left pelvic enlarged lymph node dissection was also performed simultaneously. No immediate complications such as hemorrhage, vascular injury, or infection occurred during and after surgery. One week after delivery, the patient received one cycle of chemotherapy. Four weeks later, she received chemoradiation therapy: cisplatin (25 mg/m^2^, d1–d3) combined with paclitaxel (100 mg/m^2^, d1; 60 mg/m^2^, d8) intravenously for one cycle concomitantly with radiation to pelvic and para‐aortic field‐50 Gy in 25 fractions of 2 Gy, followed by four fractions of 7 Gy of MRI‐guided brachytherapy. MRI revealed complete response (CR, RECIST1.1) 2 days after chemoradiotherapy finished (Figure 1A). SCCA concentration showed a decreased change in the course of treatment and declined to the normal range (normal cutoff: 1.5 ng/mL) after chemoradiotherapy.

The birth weight of the infant was 2.2 kg with Apgar scores of 9, 10, and 10 at 1, 5, and 10 min, respectively and normal hematological parameters at delivery. HPV test was negative in the oral cavity and anus of the infant.

Significant of current study, primary cervical cancer status, response to NACT, and disease progression were monitored by ctDNA detection of targeted NGS of 202 cancer‐related genes (GRCh37/hg19) on Illumina sequenator (NextSeq 550DX/NovaSeq 6000), meanwhile MRI and SCCA were used as safety control for monitoring cancer status as routine in this case. These data were collected at the time of initial diagnosis (1 week before the first cycle of NACT), cesarean delivery (2 weeks after the third cycle of NACT), post‐one cycle of chemotherapy postpartum (1 week later), and post‐chemoradiotherapy. To the best of our knowledge, its use in pregnant woman was not reported previously in the literature. Amniotic fluid and umbilical cord blood collected during cesarean section was also tested for ctDNA. ctDNA mean AF declined from 0.6% to 0.3% after three cycles of NACT and clearance of ctDNA was achieved after the surgery and maintained negative after chemoradiotherapy. ctDNA mutation was not identified in amniotic fluid or umbilical cord blood. The concentration of paclitaxel at the time of cesarean delivery was 35–42 ng/mL, 17–25 ng/mL, and 0 ng/mL in maternal blood, umbilical cord blood, and in amniotic fluid, respectively.

During the follow‐up of 17 months after treatment, the mother recovered well and had no clinical evidence of recurrence of cancer. During the follow‐up of 21 months, the infant grew normally, without any evidence of chemotherapy‐related side effects such as elevated creatinine levels or hearing loss. Placenta pathologic study showed no evidence of cancer metastasis.

### 2.2 Patient 2

A 37‐year‐old gravida‐2 para‐1 female presented to Zhongda Hospital at 36 weeks' gestation due to vaginal bleeding on April 14, 2022. She denied previous history of chronic diseases or gynecological tumors and had not had a cervical pap smear for almost 10 years. Pelvic exam revealed a mass of about 5 cm in diameter on the cervix. MRI of the abdomen and pelvis revealed a viable intrauterine single pregnancy, cervical mass measuring 57 × 56 × 54 mm in diameter with invasion of the anterior fornix of the vagina and left parametrium, with metastasis to the left iliac vessel lymph nodes. Final pathology confirmed moderately differentiated squamous cell carcinoma of the cervix at Stage IIIC (FIGO 2018). The SCCA value was 44.7 ng/mL (normal range 0–2.7 ng/mL), and HPV test was positive for Type 16.

Cesarean section was performed at 36 weeks' gestation, during which left pelvic enlarged lymph node dissection was also performed simultaneously. The neonate weighed 3.35 kg and Apgar scores at 1, 5, and 10 min were 5, 9, and 9, respectively. There were no immediate postoperative complications observed. The surgical pathology indicated metastasis of squamous cell carcinoma to the left pelvic lymph node, while no evidence of metastasis was found in the placenta.

NGS testing was conducted on tumor tissue and liquid biopsy samples, including maternal blood, amniotic fluid, and umbilical cord blood. Three mutations were identified in ctDNA from the mother's blood with a VAF range of 0.58%–0.96%, including FOXO1 (p.S22W), PIK3CA (p.E545K), and PIK3CA (p.E542K). Of these mutations, PIK3CA (p.E542K) was also detected in the tumor sample. Two mutations (PHO2B [p.A257_A260dup] and LATS2 [p.P471Rfs*103]) were found in the amniotic fluid but not present in the tumor sample. No ctDNA mutation was detected in umbilical cord blood.

One week postpartum, the patient underwent two cycles of chemotherapy with cisplatin (70 mg/m^2^) and paclitaxel (175 mg/m^2^) every 3 weeks. MRI revealed a partial response (PR, RECIST1.1) of local cervical lesions. The SCCA value had decreased to 6.6 ng/mL. ctDNA mean AF declined from 0.77% to 0.29%. Nine weeks after delivery with fine scar healing, the patient received chemoradiation therapy: cisplatin 40 mg/m^2^ every week intravenously for four cycles concomitantly with radiation to pelvic and para‐aortic field‐50 Gy in 25 fractions of 2 Gy, followed by four fractions of 7 Gy of MRI‐guided brachytherapy. During the course of treatment, serial analysis of ctDNA revealed a significant reduction in ctDNA levels during chemoradiotherapy (Figure 1B).

During the follow‐up of 17 months after treatment, the mother recovered well and had no clinical evidence of recurrence of cancer. During the follow‐up of 21 months, the infant grew normally without any evidence of cancer metastasis from her mother.

## 3 Discussion

We reported two patients diagnosed of cervical cancer with high tumor load, and no evidence showed the metastasis to placenta. Placental metastases enclose the direct invasion of the placental tissue by tumor cells and debatably the presence of malignant cells in the intervillous space, although the tumor cells remain within the maternal vascular space [2]. Fetal metastases are less commonly reported than placental metastases probably due to the physical and immune constraints of the placenta [11]. Without metastasis to the placenta together with the negative result of ctDNA in umbilical cord blood and amniotic fluid implied that cervical cancer may not transmit across the placental barrier and so it is safe for delayed delivery until fetal maturity in utero during pregnancy.

For pregnant women, in general, with cervical cancer who desired to continue the pregnancy, NACT beyond the first trimester was recommended to enable fetal maturation to avoid risk of spontaneous abortion, fetal death, and fetal defects [12]. Previous study on 21 patients with cervical cancer in pregnancy treated with three cycles of neoadjuvant platinum‐based chemotherapy demonstrated that platinum concentrations in the amniotic fluid were demonstrably lower than in the umbilical cord blood (11%–42% vs. 23%–65%), suggesting a placental filtration mechanism for platinum [13]. In Patient 1, undetectable concentration of paclitaxel in amniotic fluid supported the evidence of maternal safety and good obstetrical outcome of NACT administered during pregnancy. Prenatal exposure to maternal cancer with or without treatment did not impair the cognitive, cardiac, or general development of children in early childhood [14].

Traditionally, clinical assessments of cervical cancer status and progression including pelvic examination, imaging study (including computed tomography [CT], MRI), and/or even biopsy are neither sensitive nor accurate enough to determine the real‐time change of the cancer itself or its response to treatment in nonpregnant patients with rare study on pregnant patient [6]. ctDNA might serve as a noninvasive monitoring tool to avoid the imaging‐related radiation during gestation, but the data are so far still very limited [15].

In the pregnant setting, serum tumor markers (including SCCA, CA153, and CA125) showed limited specificity rate and were less reliable [16]. Compared to these protein markers in plasma, shorter half‐time of less than 2 h makes ctDNA a more accurate biomarker to reflect tumor load and therapeutic response in patient [17]. Previous study suggested that the longitudinal monitoring of ctDNA could provide unique predictive information for response to therapy in advanced cervical cancer, and might serve as an attractive alternative for patients lack of clinical sensitive markers [10]. It was also interesting that a previous study showed a higher detection rate of ctDNA in the pregnant patients with breast cancer than in their nonpregnant counterparts [18]. In this study, we first used a targeted NGS analysis of 202 cancer‐related genes in ctDNA samples from a pregnant woman with advanced cervical cancer. Our study suggested the ctDNA abundance decreased along with tumor regression which was confirmed by traditional using SCCA and MRI monitoring for safety control. This result was consistent with previous studies that ctDNA dynamics may indicate if the disease will response to the treatment or not [10]. In Patient 1, mutations in maternal ctDNA sample at baseline were discovered, including the most common genes in cervical cancer PIK3CA, PTEN, and MAPK1. Variations in MAPK1 were detected exclusively in squamous tumors [19] and could be a compensatory diagnostic biomarker to distinguish with adenocarcinomas in our case. With plasma of 150 patients with primary invasive cervical cancer, Chung TKH et al. found that mutation in the PIK3CA gene p.E542K and p.E545K were significantly associated with both reduced disease‐free survival and overall survival [20]. Variation of PIK3CA p.E545K was detected in Patient 2, which may imply a more frequent follow‐up visit in this patient.

## 4 Conclusion

Our case series provided a practical management of advanced cervical cancer during pregnancy. Monitoring maternal ctDNA during pregnancy might serve as an auxiliary tool to monitor cervical cancer status and its response to NACT when definite treatment delay is decided. However, the study had limitation of the lack of long‐term maternal and neonatal outcomes as well as the lack of evaluation of utility of ctDNA during pregnancy. Further study is needed to confirm its value in management of pregnant women with cervical or other malignances.

## Author Contributions

**Xiaoyu Zhang:** conceptualization, project administration, data curation, writing – original draft. **Hong Yu:** project administration, supervision, data curation. **Kai Chen:** conceptualization, data curation, writing – review and editing. **Bo Ding:** investigation, methodology, writing – review and editing. **Yang Shen:** conceptualization, data curation, project administration, supervision, writing – review and editing.

## Ethics Statement

The study was approved by the Institutional Ethics Committee for Clinical Research of Zhongda Hospital, Affiliated to Southeast University, approval number [2022ZDSYLL373‐P01]. Written informed consent was obtained from the patient for the publication of the report and any associated medical images. A copy of the written consent is available for review by the editor‐in‐chief of this journal on request.

## Conflicts of Interest

The authors declare no conflicts of interest.

## Data Availability

The data that support the findings of this study are available from the corresponding author upon reasonable request.

## References

[1] Z. He , C. Xie , X. Qi , Z. Hu , and Y. He , “The Effect of Preserving Pregnancy in Cervical Cancer Diagnosed During Pregnancy: A Retrospective Study,” BMC Womens Health 22, no. 1 (2022): 314. 35879712 10.1186/s12905-022-01885-w PMC9317436

[2] M. La Russa and A. R. Jeyarajah , “Invasive Cervical Cancer in Pregnancy,” Best Practice & Research. Clinical Obstetrics & Gynaecology 33 (2016): 44–57. 26586539 10.1016/j.bpobgyn.2015.10.002

[3] G. Ferrandina , A. Ercoli , A. Fagotti , et al., “Completion Surgery After Concomitant Chemoradiation in Locally Advanced Cervical Cancer: A Comprehensive Analysis of Pattern of Postoperative Complications,” Annals of Surgical Oncology 21, no. 5 (2014): 1692–1699. 24407316 10.1245/s10434-013-3471-y

[4] R. Fruscio , A. Villa , S. Chiari , et al., “Delivery Delay With Neoadjuvant Chemotherapy for Cervical Cancer Patients During Pregnancy: A Series of Nine Cases and Literature Review,” Gynecologic Oncology 126, no. 2 (2012): 192–197. 22555106 10.1016/j.ygyno.2012.04.027

[5] C. Ricci , G. Scambia , and R. De Vincenzo , “Locally Advanced Cervical Cancer in Pregnancy: Overcoming the Challenge. A Case Series and Review of the Literature,” International Journal of Gynecological Cancer 26, no. 8 (2016): 1490–1496. 27575627 10.1097/IGC.0000000000000795

[6] M. H. D. Neumann , S. Bender , T. Krahn , and T. Schlange , “ctDNA and CTCs in Liquid Biopsy—Current Status and Where We Need to Progress,” Computational and Structural Biotechnology Journal 16 (2018): 190–195. 29977481 10.1016/j.csbj.2018.05.002 PMC6024152

[7] P. Cafforio , R. Palmirotta , D. Lovero , et al., “Liquid Biopsy in Cervical Cancer: Hopes and Pitfalls,” Cancers 13, no. 16 (2021): 3968. 34439120 10.3390/cancers13163968 PMC8394398

[8] L. M. Charo , R. N. Eskander , R. Okamura , et al., “Clinical Implications of Plasma Circulating Tumor DNA in Gynecologic Cancer Patients,” Molecular Oncology 15, no. 1 (2021): 67–79. 32881280 10.1002/1878-0261.12791 PMC7782073

[9] E. Kilgour , D. G. Rothwell , G. Brady , and C. Dive , “Liquid Biopsy‐Based Biomarkers of Treatment Response and Resistance,” Cancer Cell 37, no. 4 (2020): 485–495. 32289272 10.1016/j.ccell.2020.03.012

[10] X. Tian , D. Ge , F. Zhang , et al., “Dynamic Analysis of Circulating Tumor DNA to Predict Prognosis and Monitor Therapeutic Response in Metastatic Relapsed Cervical Cancer,” International Journal of Cancer 148, no. 4 (2021): 921–931. 33113150 10.1002/ijc.33362

[11] K. V. Calsteren , R. Verbesselt , R. Devlieger , et al., “Transplacental Transfer of Paclitaxel, Docetaxel, Carboplatin, and Trastuzumab in a Baboon Model,” International Journal of Gynecological Cancer 20, no. 9 (2010): 1456–1464. 21307819 10.1111/IGC.0b013e3181fb18c8

[12] Y. Song , Y. Liu , M. Lin , B. Sheng , and X. Zhu , “Efficacy of Neoadjuvant Platinum‐Based Chemotherapy During the Second and Third Trimester of Pregnancy in Women With Cervical Cancer: An Updated Systematic Review and Meta‐Analysis,” Drug Design, Development and Therapy 13 (2018): 79–102. 30587930 10.2147/DDDT.S186966 PMC6304076

[13] C. Kohler , P. Oppelt , G. Favero , et al., “How Much Platinum Passes the Placental Barrier? Analysis of Platinum Applications in 21 Patients With Cervical Cancer During Pregnancy,” American Journal of Obstetrics and Gynecology 213, no. 206 (2015): e1–e5. 10.1016/j.ajog.2015.02.022 25731691

[14] F. Amant , T. Vandenbroucke , M. Verheecke , et al., “Pediatric Outcome After Maternal Cancer Diagnosed During Pregnancy,” New England Journal of Medicine 373, no. 19 (2015): 1824–1834, 10.1056/NEJMoa1508913. 26415085

[15] S. A. Cohen , A. Kasi , N. Hook , et al., “The Utility of Circulating Tumor DNA (ctDNA) Monitoring in Cancer Patients who Are Pregnant or Planning to Become Pregnant,” Case Reports in Obstetrics and Gynecology 2022 (2022): 9412201. 35342654 10.1155/2022/9412201 PMC8941578

[16] S. N. Han , A. Lotgerink , M. M. Gziri , K. van Calsteren , M. Hanssens , and F. Amant , “Physiologic Variations of Serum Tumor Markers in Gynecological Malignancies During Pregnancy: A Systematic Review,” BMC Medicine 10 (2012): 86. 22873292 10.1186/1741-7015-10-86 PMC3425318

[17] F. Cheng , L. Su , and C. Qian , “Circulating Tumor DNA: A Promising Biomarker in the Liquid Biopsy of Cancer,” Oncotarget 7, no. 30 (2016): 48832–48841. 27223063 10.18632/oncotarget.9453 PMC5217053

[18] L. Lenaerts , H. Che , N. Brison , et al., “Breast Cancer Detection and Treatment Monitoring Using a Noninvasive Prenatal Testing Platform: Utility in Pregnant and Nonpregnant Populations,” Clinical Chemistry 66, no. 11 (2020): 1414–1423. 33141904 10.1093/clinchem/hvaa196

[19] Cancer Genome Atlas Research Network, Albert Einstein College of Medicine, Analytical Biological Services , et al., “Integrated Genomic and Molecular Characterization of Cervical Cancer,” Nature 543, no. 7645 (2017): 378–384. 28112728 10.1038/nature21386 PMC5354998

[20] T. K. H. Chung , T. H. Cheung , S. F. Yim , et al., “Liquid Biopsy of PIK3CA Mutations in Cervical Cancer in Hong Kong Chinese Women,” Gynecologic Oncology 146 (2017): 334–339. 28587748 10.1016/j.ygyno.2017.05.038

